# Regulation of Wound Healing by Granulocyte-Macrophage Colony-Stimulating Factor after Vocal Fold Injury

**DOI:** 10.1371/journal.pone.0054256

**Published:** 2013-01-25

**Authors:** Jae-Yol Lim, Byung Hyune Choi, Songyi Lee, Yun Ho Jang, Jeong-Seok Choi, Young-Mo Kim

**Affiliations:** 1 Department of Otorhinolaryngology-Head and Neck Surgery, Inha University School of Medicine, Incheon, Republic of Korea; 2 Translational Research Center, Inha University School of Medicine, Incheon, Republic of Korea; 3 Division of Biomedical and Bioengineering Sciences, Inha University School of Medicine, Incheon, Republic of Korea; Beijing Institiute of Otolaryngology, China

## Abstract

**Objectives:**

Vocal fold (VF) scarring remains a therapeutic challenge. Granulocyte-macrophage colony-stimulating factor (GM-CSF) facilitates epithelial wound healing, and recently, growth factor therapy has been applied to promote tissue repair. This study was undertaken to investigate the effect of GM-CSF on VF wound healing *in vivo* and *in vitro*.

**Methods:**

VF scarring was induced in New Zealand white rabbits by direct injury. Immediately thereafter, either GM-CSF or PBS was injected into the VFs of rabbits. Endoscopic, histopathological, immunohistochemical, and biomechanical evaluations of VFs were performed at 3 months post-injury. Human vocal fold fibroblasts (hVFFs) were cultured with GM-CSF. Production of type I and III collagen was examined immunocytochemically, and the synthesis of elastin and hyaluronic acids was evaluated by ELISA. The mRNA levels of genes related to ECM components and ECM production-related growth factors, such as HGF and TGF-ß1, were examined by real time RT-PCR.

**Results:**

The GM-CSF-treated VFs showed reduced collagen deposition in comparison to the PBS-injected controls (*P*<0.05). Immunohistochemical staining revealed lower amounts of type I collagen and fibronectin in the GM-CSF-treated VFs (*P*<0.05 and *P*<0.01, respectively). Viscous and elastic shear moduli of VF samples were significantly lower in the GM-CSF group than in the PBS-injected group (*P*<0.001 and *P*<0.01, respectively). Mucosal waves in the GM-CSF group showed significant improvement when compared to the PBS group (*P* = 0.0446). GM-CSF inhibited TGF-β1-induced collagen synthesis by hVFFs (*P*<0.05) and the production of hyaluronic acids increased at 72 hours post-treatment (*P*<0.05). The expressions of HAS-2, tropoelastin, MMP-1, HGF, and c-Met mRNA were significantly increased by GM-CSF, although at different time points (*P*<0.05).

**Conclusion:**

The present study shows that GM-CSF offers therapeutic potential for the remodeling of VF wounds and the promotion of VF regeneration.

## Introduction

Vocal folds (VFs) are vital for voice production via aerodynamically driven oscillation of pliable, layered VF mucosa. Harmonious oscillation of VF mucosa, which is important for the maintenance of voice quality, is mainly attributed to the biomechanics of the layered structure of VFs. The biomechanical properties of VF layers are primarily characterized by their extracellular matrix (ECM) composition and organization [1]. VF injury caused by vocal abuse, surgery, or inflammation commonly results in VF scarring, which disrupts the layered structure of VFs, alters the biomechanical properties of layered VFs, and possibly leads to intractable dysphonia [2].

A number of recent studies have examined changes in ECM synthesis and distribution in VFs and found excessive collagen deposition, low elastin density and disorganization, as well as a low HA level, in diverse animal VF scar models [3]–[7]. Regarding the biomechanics of VFs after scarring, elastic shear modulus and dynamic viscosity have been reported to be higher for scarred VFs, and suggested to be due to alterations in ECM composition and organization [3], [6]. Accordingly, the remodeling of ECM composition and organization by encouraging VF fibroblasts to synthesize favorable ECM components during the wound healing phase has been the focus of a number of studies undertaken to prevent or mitigate scarring.

Recently, a variety of bioactive factors have been investigated in regards to their ability to promote VF wound healing. A few authors have shown that the local administration of growth factors, including hepatocyte growth factor (HGF) and basic fibroblast growth factor (bFGF), in injured VFs leads to improved histologic and biomechanical properties in *in vivo* animal models [8], [9]. Such growth factors are considered to affect VF fibroblasts during the wound healing process, enhancing ECM synthesis and deposition [10]. However, the complex roles played by many growth factors and cytokines involved in the inflammatory phase of VF wound healing have not been fully elucidated.

Granulocyte-macrophage colony-stimulating factor (GM-CSF) is a multipotent cytokine that is synthesized by many cell types, including macrophages, lymphocytes, fibroblasts, and endothelial cells, and causes the chemotaxis of inflammatory cells to wound sites. Additionally, GM-CSF stimulates the proliferation and differentiation of hematopoietic progenitor cells, increases neovascularization, enhances epithelial regeneration, and plays a complex tissue-dependent role in fibrosis [11]–[13]. Recently, topically applied GM-CSF has been suggested to play an important role in cutaneous wound repair. A series of animal experiments and clinical studies have demonstrated a beneficial effect of GM-CSF for the promotion of wound healing of burns and chronic ulcers [12], [14]. In one of our previous studies, exogenous GM-CSF was also shown to inhibit glial scar formation in a spinal cord injury model in rats [15]. However, to date, no previous study has examined the effect of GM-CSF on VF wound healing; more specifically, studies of GM-CSF on ECM modulation and tissue repair are scarce.

Taking these studies together, we hypothesized that GM-CSF would promote wound remodeling following VF injury, and that the local administration of GM-CSF would improve VF regeneration. To prove this hypothesis and to assess the potential of GM-CSF as a novel therapeutic candidate for VF wound healing, we investigated the effects of injection of GM-CSF on VF wound healing in a rabbit model and investigated the mechanisms involved using *in vitro* cultured human VF fibroblasts (hVFFs). Accordingly, functional, macro- and micromorphological evaluations were performed *in vivo*. For the *in vitro* model, the primary outcome measures were morphology, proliferation, and the production of ECM components, such as collagen, elastin, and hyaluronic acid (HA). In addition, we assessed the expressions of genes related to ECM components and ECM production-related growth factors, such as HGF and TGF-ß1.

## Materials and Methods

### Ethics statement

This study was approved by the Animal Ethics Committee of The Inha University Hospital (Permit Number: 111031-114), and animal care was strictly provided according to established institutional guidelines. All surgery was performed under anesthesia by premedication with xylazine (5 mg/kg) and an intramuscular injection of 15 mg/kg of zolazepam, making every effort to minimize suffering.

### Animal experiments

Selection of an animal model depends on the structural characteristics of the animal's VFs, as well as other practical considerations. Rabbit models have been widely used in VF scar research because of an appropriate VF size for function measurement, as well as due to similarities in the layered structure and ECM components of rabbit VFs with human VFs [16]. For the experiments, 30 New Zealand white rabbits weighing 3.1–3.6 kg were used. The animals were randomly divided into three groups of 10 rabbits: an uninjured group (normal), an injured and phosphate-buffered saline (PBS) treated group (scar control), and an injured and GM-CSF treated group (experimental group). The animals were pre-medicated subcutaneously with 0.05 mg/kg of glycopyrrolate and then anesthetized. The larynx was visualized using a pediatric laryngoscope (Karl Storz, Tuttlingen, Germany) and a surgical operating microscope (Carl Zeiss Ltd, Welwyn Garden City, UK). Unilateral VF injury was induced in six animals from each group as previously described; the method involved excising VF epithelium and lamina propria using a sickle knife and microcup forceps [17]. Contralateral VFs were used as a control. Bilateral VF injuries were administered in four animals of each group for rheological evaluation.

Immediately after injury, 50 µL of rhGM-CSF (1 mg/mL in saline) was directly administrated into VFs in the experimental group. In the scar control group, 50 µL of PBS was injected. A Hamilton syringe with a 25 G needle was used to inject PBS or GM-CSF to VFs under direct vision using a pediatric laryngoscope and surgical operating microscope.

### In vivo assessment

#### Macroscopic evaluation and high speed digital imaging

At 1 and 3 months post-injury, an endoscopic evaluation was performed in all three groups and scar formation on VFs was assessed macroscopically. Two larynges were then excised post-euthanasia for *ex vivo* evaluation of the mucosal wave. Briefly, the larynx was mounted on a table, through which airflow was passed from an airflow generator below the table to the larynx to generate vocal fold vibrations. All supraglottic structures were removed for better visualization and VFs were closed by suturing the vocal processes of arytenoid cartilages. Symmetry was maintained across the mid sagittal plane using arytenoid micromanipulators, such that both VFs were aligned. Expiratory airflow was artificially generated by allowing a certain volume of room air to flow constantly. Mucosal waves were recorded using a High speed digital imaging system (MegaSpeed HHCX6, Canadian Photonic Labs INC, Minnedosa, CA) in gray scale at an image size of 512×512 pixels. Images of duration with 0.2 seconds were captured at 5000 frames per second (fps). A single line in mid-coronal glottal plane from high speed digital images was selected to produce kymographs. The amplitudes of mucosal waves were evaluated by measuring the pixels of oscillation of VF upper lips between the open phase and closed phase on kymographs using image analysis software (Metamorph, Molecular Devices Corporation, Sunnyvale, CA), and were normalized relative to those of contralateral normal VF. During the examination, VFs were maintained wet by dripping saline solution at 37°C

#### Functional rheometric evaluation

Three months post-injury, four larynges in each group were removed post-euthanasia. VFs without muscles and other supra- and subglottic connective tissues were meticulously dissected under magnified vision using a surgical operating microscope. Each VF sample was approximately 5 to 6 mm in length and 2 to 3 mm in depth. The eight dissected VFs were stored in normal saline at 37°C for a couple of hours until viscoelastic measurements were undertaken.

A strain-controlled rheometer (ARES-LS, TA Instruments, New Castle, Delaware, USA) was used to measure the viscoelasticity of the dissected VFs. A sample was placed between the 2 parallel plates of the rheometer. The rotating lower plate included a temperature control system and a steady or oscillatory shear deformation upon the samples. A piezoelectric force transducer of the upper plate with measurement geometry recorded the shear stresses resulting from steady or oscillatory shear deformation. The diameter of the upper plate used for VF tissue samples was 3 mm, and inter-plate gap size was at 0.8–1.2 mm to allow full contact between samples and the upper plate. The temperature of the lower plate was set at 37°C, and samples were maintained in a humidified environment (RH 100%).

A dynamic frequency sweep test at frequencies ranging from 0.1 to 100 rad/s was used to apply oscillatory shear deformation to the samples. Sample linear viscoelastic conditions were measured using a series of dynamic strain sweep tests prior to frequency sweep testing. TA Orchestrator software (ARES-LS, TA Instruments, New Castle, Delaware, USA) was used to calculate shear stresses, shear strains, and shear strain rates. Viscoelastic parameters, such as elastic modulus (*G′*) and viscous modulus (*G″*), were calculated as functions of applied frequency according to the theory of linear viscoelasticity. Dependence of viscoelastic parameters on frequency can be parameterized by the power law, in which the coefficient ‘*a*’ indicates magnitude and the constant ‘*b*’ indicates slope on a log-log scale.

#### Histopathological and immunohistochemical examination

Four larynges in each group were harvested for histological analysis post-euthanasia at 3 months post-injury. Specimens were embedded in paraffin blocks and embedded tissue samples were sectioned at 4 µm using a microtome along the coronal axis of the larynx and stained with; hematoxylin and eosin (H-E), Masson's trichrome (for collagen), Verhoeff's van Gieson (for elastic fibers), or alcian blue (for total glycosaminoglycans) using standard pathology department protocols.

For immunohistochemical examinations of collagen type I and fibronectin, paraffin sections of excised larynges were deparaffinized in xylene, rehydrated in ethanol, and washed with PBS. Thereafter, nonspecific binding was blocked with a blocking solution containing 1% bovine serum albumin. Slides were incubated overnight at 4°C with a primary antibody against collagen type I (1∶100 dilution, AB Biotec) and fibronectin (1∶50 dilution, Abcam). Secondary Alexa fluor 555-conjugated anti-mouse IgG antibodies (1∶500 dilutions, Cell Signaling) were then applied to the slides for 1 hour in the dark at room temperature, followed by 4′,6-diamidino-2-phenylindole dihydrochloride (DAPI; Vector Labs, H-1500) treatment for 3–5 minutes to stain cell nuclei. All experiments included a slide not treated with primary antibody as a negative control. Three sections were prepared for each VF, and 12 slides in total were analyzed using a confocal laser-scanning microscope. A blinded examiner measured the ECM contents by calculating pixel areas using MetaMorph software (Molecular Devices Corporation, Sunnyvale, CA, USA).

### Immortalized hVFF cell lines

The immortalized hVFF cell lines (cell line #A8P3) were kindly provided by Susan L. Thibeault at the University of Wisconsin. Full immortalization details and characterization of this cell line are described elsewhere [18]. Immortalized hVFFs showed similar morphological features and ECM gene expressions beyond passage 25. For this experiment, hVFFs obtained between the 5^th^ and 6^th^ passages were used.

### In vitro assessments

#### Cell morphology and proliferation assay

The morphology of hVFFs was observed using phase contrast microscopy. Cell proliferation was assessed in triplicate using a Cell Counting Kit-8 (CCK-8; Dojindo, Gaithersburg, MD), according to the manufacturer's instructions. Aliquots of hVFF cells (100 µL/3×10^3^ cells) cultured in 96-well plates were serum-starved overnight and then treated with serum-free medium containing 0, 10, 100, or 500 ng/mL of GM-CSF for 12, 24, or 72 hours. The CCK-8 reagent (10 µL) was then added to each well 1 h before completing the incubation. Absorbance at 450 nm was measured using a microplate reader.

#### Biochemical analyses

The production of insoluble elastin was quantified biochemically. Elastin amounts in hVFFs were measured using a Fastin elastin assay kit (Biocolor Ltd, Carrickfergus, UK). Since the Fastin assay quantified only soluble α-elastin, all samples were converted from the native hydrophobic elastin into a water soluble derivative. To extract α-elastin, samples were heated at 100°C for 1 h period with 0.25 M oxalic acid. Solubilized proteins containing elastin extract were precipitated overnight by adding Fastin precipitating reagent. Collected pellets (12,000 rpm for 10 min) were mixed with Fastin dye reagent, which contained 5,10,15,20-tetraphenyl-21H, 23H-propine tetrasulfonate (TPPS) in a citrate-phosphate buffer for 90 min. The elastin-dye complex obtained was precipitated (12,000 rpm for 10 min), mixed with dye dissociation reagent for 10 min, and absorbance of the recovered dye, α-elastin standards, and blanks were measured at 513 nm using a microplate reader.

Quantitation of HA levels in culture media was performed using an enzyme-linked immuno assay (ELISA). HA in media was measured in triplicate using an ELISA kit (Echelon Biosciences, Salt Lake City, UT, USA). Briefly, the HA detector was mixed with HA standard and culture media and incubated for 1 hour at 37°C, after which samples were transferred to a detection plate. After incubation for 30 min at 4°C working enzyme was added to the detection plate and samples were incubated in the dark at room temperature. Finally, absorbance was measured at 405 nm.

#### Immunocytochemical analyses

The synthesis of types I and III collagen from the hVFFs was stimulated by TGF-ß1 and then analyzed by immunocytochemical staining. Briefly, near-confluent hVFFs were serum-starved overnight and treated with 10 ng/mL of TGF-ß1 with or without 500 ng/mL of GM-CSF for 5 days. Cultured hVFFs were then washed with PBS, fixed in 4% paraformaldehyde (PFA) for 15 min, and permeabilized in PBS containing 0.2% Triton X-100 (Sigma-Aldrich, St. Louis, MO, USA) for 10 min. Nonspecific binding was blocked in a blocking solution containing 2% bovine serum albumin, and slides were incubated with primary antibody against type I or III collagen (1∶100 dilution, AB Biotec, San Diego, CA, USA) overnight at 4°C. Secondary Alexa fluor 488-conjugated anti-rabbit IgG antibodies (1∶500 dilutions, Invitrogen, Carlsbad, CA, USA) were then applied to the slides for 1 hour in the dark at room temperature, and 4′,6-diamidino-2-phenylindole, dihydrochloride (DAPI; Vector Labs, H-1500) was applied for 3–5 min to stain cell nuclei. All experiments included a slide not treated with primary antibody as a negative control. Slides were analyzed using laser-scanning confocal microscopy (Olympus FV1000, Japan) at a magnification of ×400. Type I and III collagen production was measured by calculating pixels in areas of positive staining using MetaMorph software.

#### Transcriptional evaluation

Total RNA was harvested from hVFF cells using Trizol (Invitrogen, Carlsbad, CA) according to the manufacturer's directions. Total RNA concentrations were measured spectrophotometrically, and RNA amounts were assessed by gel electrophoresis. One microgram of total RNA was transcribed into complementary DNA using a PrimeScriptTM RT reagent kit (Takara, Japan). Real-time reverse transcriptase-polymerase chain reaction (RT-PCR) was carried out in 20 µl of reagents (SYBR Premix Ex Taq™ II, Takara #RR081A) containing cDNA (1 µl) and the specific primers for each gene (detailed in Table 1). RT-PCR was performed using the StepOnePlus Real-Time PCR system (Applied Biosystems, Foster City, CA) over 40 cycles of; denaturation at 95°C for 10 sec, annealing at 55°C for 25 sec, and extension at 72°C for 30 sec. After amplification, reaction efficiencies were determined using melting curves constructed from purified PCR products and mRNA levels in tissue samples were quantified. Negative controls containing water instead of template and initial mRNA were included in each run to rule out the contamination of DNA and genomic DNA. The housekeeping gene of glyceraldehyde-3-phosphate dehydrogenase (GAPDH) was used as an internal control. Relative gene expressions (fold-changes) were calculated using the 2-ΔΔCT method.

**Table 1:** Primer sequences used for real time PCR.

Genes	Forward primer sequences	Reverse primer sequences
HA synthease-2	5′-GGGGGAGATGTCCAGATTTT-3′	5′-ATGCACTGAACACACCCAAA-3′
TGF-β1	5′-ACAATTCCTGGCGATACCTCA-3′	5′-GGCGAAAGCCCTCAATTTC-3′
Fibronectin	5′-CAATCCAGAGGAACAAGCATGTCTC-3′	5′-GCTTTCCTATTGATCCCAAACCAAA-3′
Tropoelastin	5′-CGAACTTTGCTGCTGCTTTAG-3′	5′-GTGTATACCCAGGTGGCGTG-3′
HGF	5′-CCTAGATCTTTCCAGTTAATCACACAAC-3′	5′-TTCGGAGTCAGTGCCTAAAAGAG-3′
c-Met	5′-TTAAAGGAGACCTCACCATGTAATC-3′	5′-CCTGATCGAGAAACCACAACCT-3′
MMP-1	5′-GGGAGATCATCGGGACAACTC-3′	5′-GGGCCTGGTTGAAAAGCA-3′
MMP-2	5′-TTCCTGGGCAACAAATATGAGA-3′	5′-TGGTCGCACACCACATCTTT-3′
Collagen I	5′-CAGCCGCTTCACCTACAGC-3′	5′- TTTTGTATTCAATCACTGTCTTGCC-3′
Collagen III	5′- ACACGTTTGGTTTGGAGAGTCC-3′	5′-CTGCACATCAACGACATCTTCAG -3′
GAPDH	5′-ATGGGGAAGGTGAAGGTCG-3′	5′-TAAAAGCAGCCCTGGTGACC-3′

### Statistics

All statistical analyses were conducted using the Graph Pad Prism 5 package (GraphPad Software Inc., La Jolla, CA). The Mann-Whitney test was used to determine the significances of differences between two groups, and the Kruskal-Wallis followed by the Dunns' post hoc test was used to compare values of the three groups. Rheological data were analyzed using the two-way ANOVA by post hoc test (Bonferroni test). Statistical significance was accepted for p values<0.05.

## Results

### Macroscopic and functional evaluation

**Figure 1:**
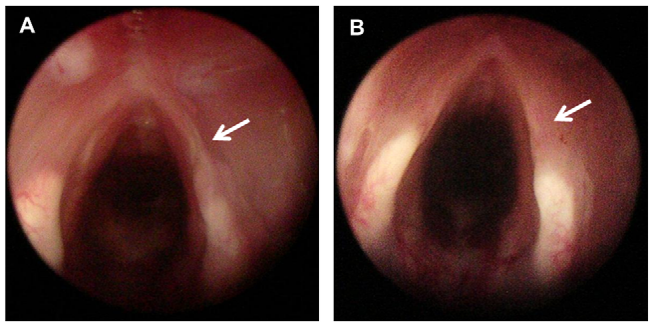
Macroscopic findings of scarred VFs at 3 months after PBS or GM-CSF treatment. Morphological improvement was observed in (B) GM-CSF treated VFs, which showed smaller fibrotic bands (arrow). In contrast, scar bands (arrow) remained prominent in (A) PBS-treated control VFs.

Macroscopic evaluations were performed at 1 and 3 months post-VF injury. Endoscopic findings revealed that fibrotic scarring was not prominent in GM-CSF-treated VFs, whereas scar bands remained prominent at 3 months post-injury in PBS-treated control VFs (Fig. 1).

**Figure 2:**
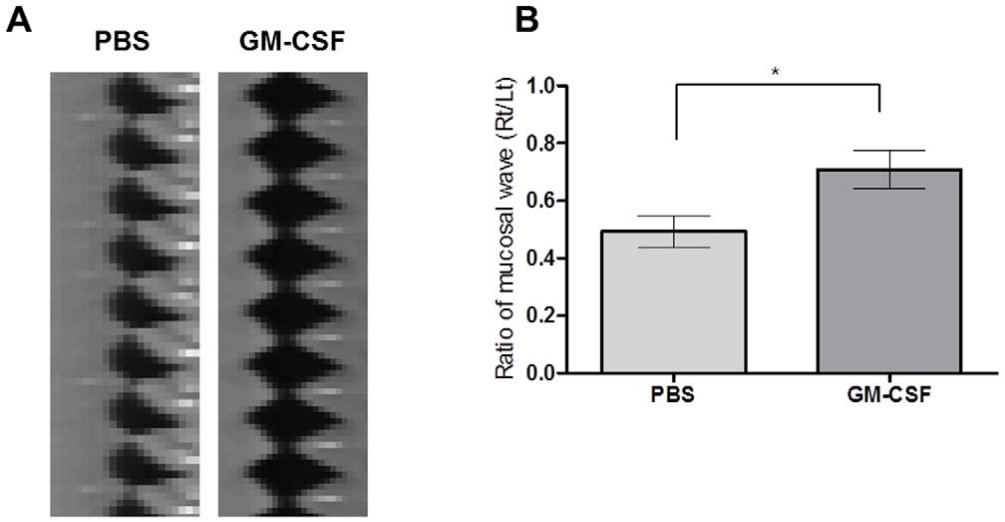
Kymographs from high-speed digital images. (A) The amplitudes of mucosal waves (MW) of VFs were measured in triplicate by measuring the pixels of oscillation of upper lips between the open phase and closed phase on high-speed digital images using image analysis software. (B) The amplitude ratio of MWs in injured right VFs relative to normal contralateral VFs was measured quantitatively. The normalized MW was significantly greater in the GM-CSF group than in the PBS group (n = 6, *P* = 0.0446).

For functional evaluations, the amplitudes of VF mucosal waves were measured by kymography from high-speed digital images (Fig. 2A). The ratio of the amplitudes of mucosal waves of injured right VFs versus normal contralateral VFs were significantly higher in the GM-CSF group than in the PBS group (*P* = 0.0446, Fig. 2B).

**Figure 3:**
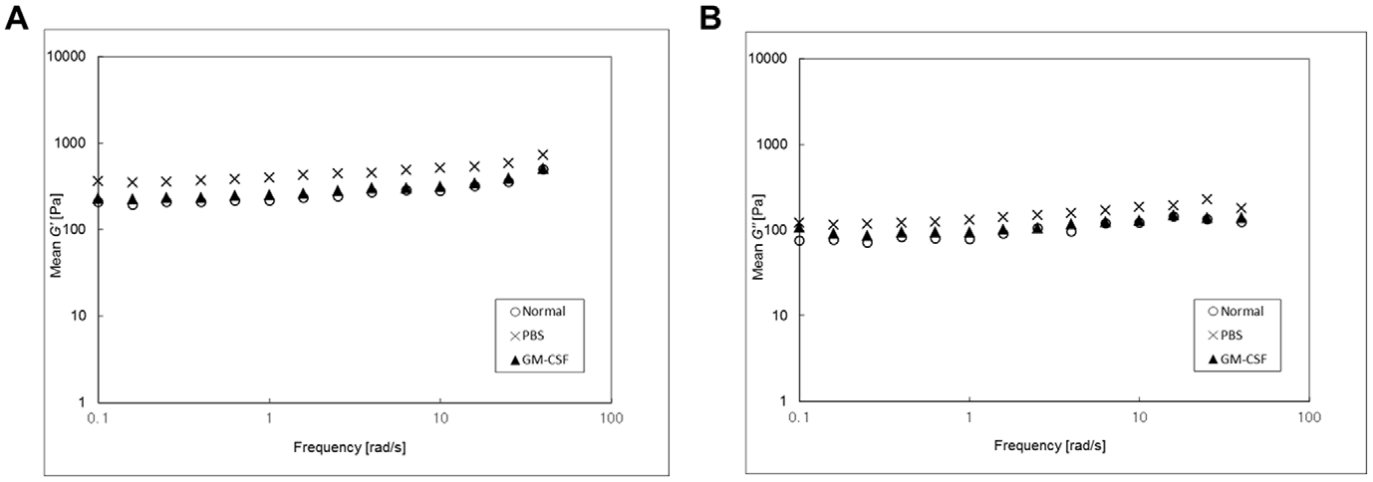
Functional rheometric evaluation at 3 months after injury: mean elastic moduli (*G′*) and viscous moduli (*G″*) were plotted on a log-log scale as a function of frequency. (A) The *G′* values of VF samples were significantly lower in the GM-CSF group (▴) than in the injured PBS group (X) (n = 8, *P*<0.001). (B) The *G″* values of tissue samples were also significantly lower in the GM-CSF group (▴) than in the PBS treated group (X) (n = 8, *P*<0.01). Both of the values in the GM-CSF group were quite a similar to those of normal VF samples (○).

Viscoelastic properties of VFs were plotted on a log-log scale as a function of the frequency (Fig. 3). The PBS group had significantly higher *G′* and *G″* values than the non-injured control group (*P*<0.001 or both of *G′* and *G″*) and the GM-CSF group (*P*<0.001 and *P*<0.01, respectively), indicating that GM-CSF treated VFs were more fluidic and less stiff than PBS treated VFs. Relations between the viscoelastic properties and frequency were described by *G′* or *G″* = *a*x*^b^*, where ‘*a*’ was equal to the magnitude and ‘*b*’ the slope on the log-log scale. Curve fitting results are shown in Table 2.

**Table 2:** Results of curve fitting by the power law of the plots between mean elastic modulus (*G′*) and mean viscous modulus (*G″*) and applied frequencies for tissue samples of VFs.

*G′ = a*x*^b^*	*a* (Pa•s)	*b*	*R^2^*
Normal	235.07±37	0.123	0.752
PBS	415.82±157.3	0.111	0.883
GM-CSF	265.13±92.83	0.118	0.823

*G″* = *a*x^b^			
Normal	94.83±69.24	0.125	0.51
PBS	156.61±67.78	0.122	0.817
GM-CSF	102.94±32.74	0.08	0.508

### Histopathology and immunohistochemistry

**Figure 4:**
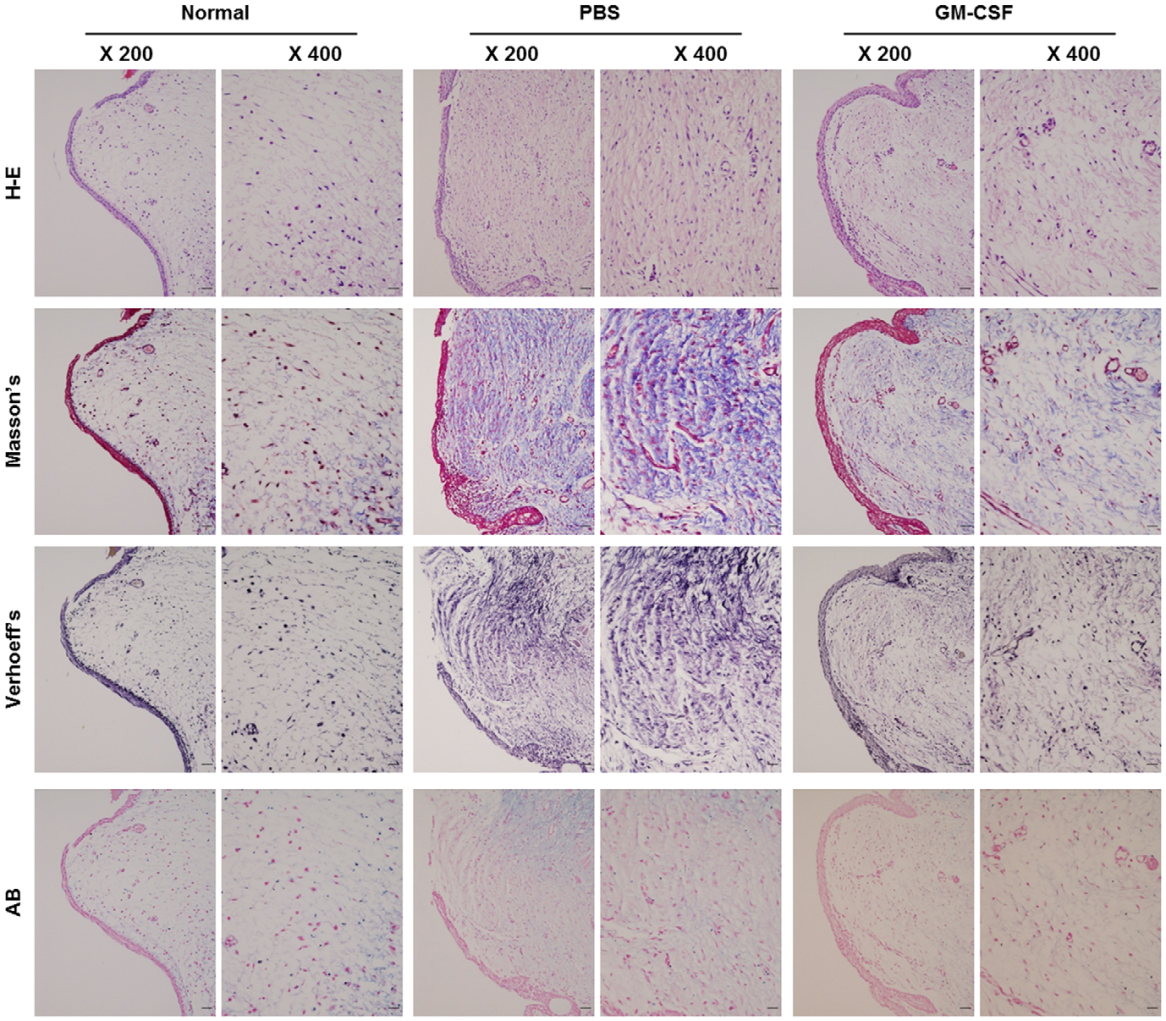
Histopathology of VFs at 3 months post-injury in the PBS and GM-CSF groups. H-E and Masson's trichrome staining showed less collagen deposition in GM-CSF-treated VFs. Verhoeff's van Gieson staining showed less disorganized and less tangled elastin in the GM-CSF-treated VFs, and VFs in the GM-CSF group appeared to be richer in glycosaminoglycans in the alcian blue staining. Scale bar = 50 µm in 200X, 100 µm in 400X.

H-E staining and Masson's trichrome staining showed that GM-CSF treatment exerted favorable effects. GM-CSF reduced collagen deposition (Fig. 4). Verhoeff's staining revealed that elastic fibers in the GM-CSF group tended to be less disorganized and less tangled. Alcian blue staining revealed that VFs in the GM-CSF group tended to exhibit higher glycosaminoglycan levels than VFs in the PBS group.

**Figure 5:**
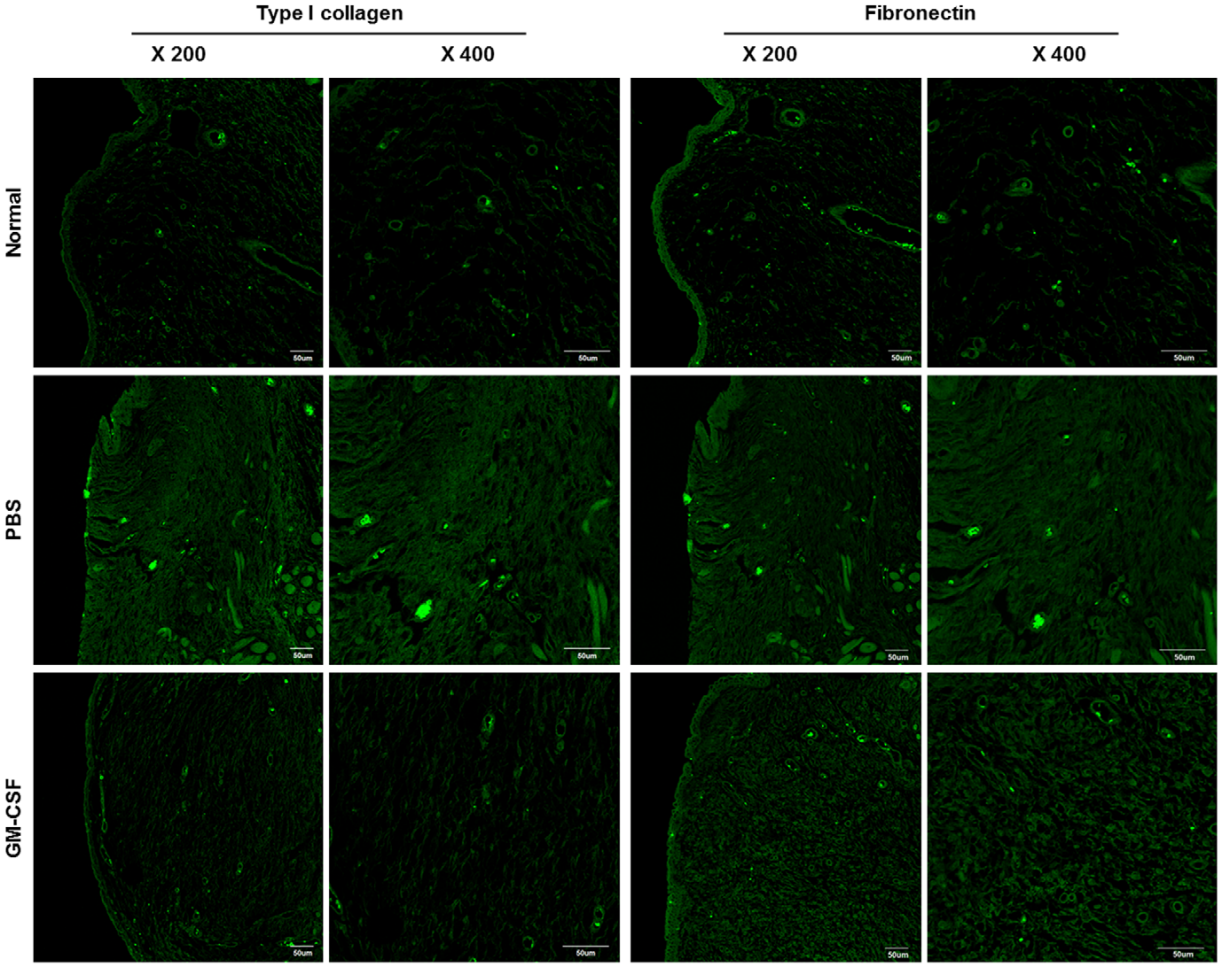
Immunohistochemical staining of type I collagen and fibronectin in the VFs of GM-CSF and PBS groups at 3 months post-injury. Immunohistochemical analysis of VFs demonstrated that the type I collagen and fibronectin levels, shown in the green fluorescence, were lower in the GM-CSF-treated VFs than in the PBS-treated VFs. Scale bar = 50 µm.

**Figure 6:**
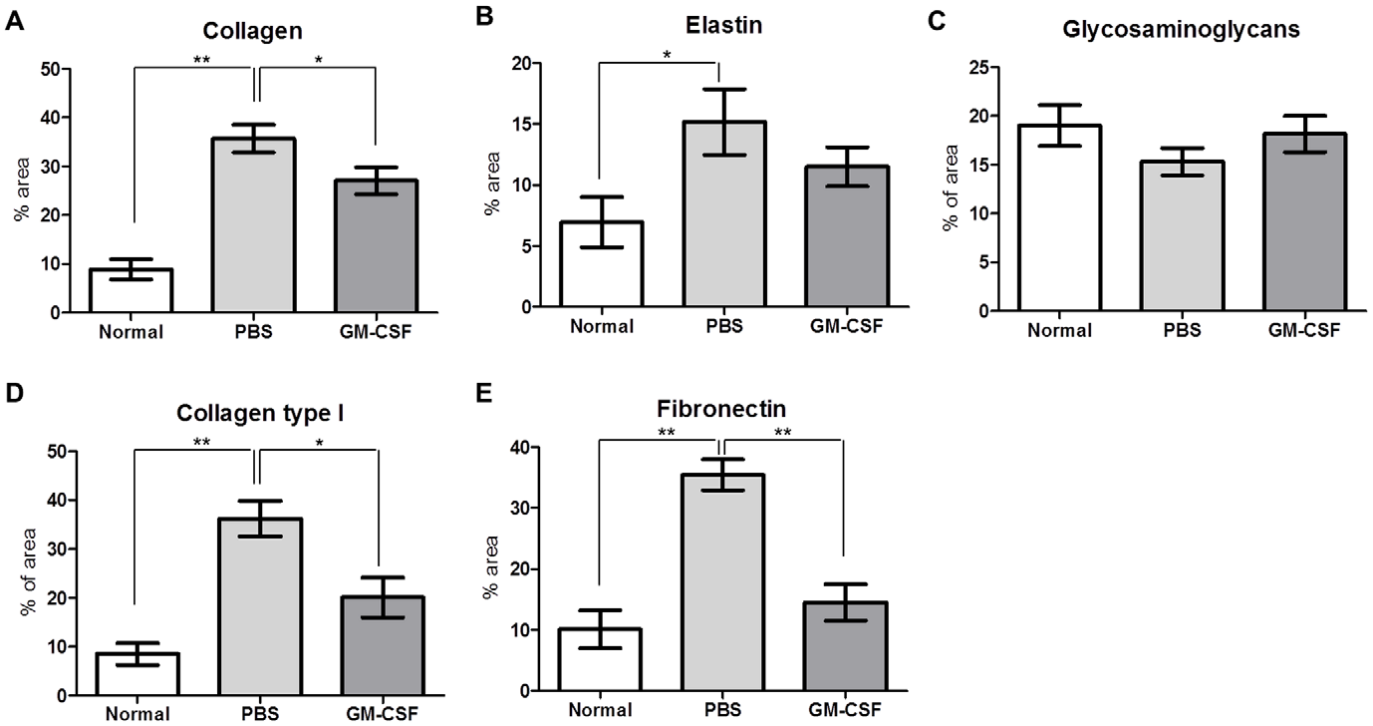
Densitometry findings for ECM contents in regenerated VFs. The histochemical or immunohistochemical images were used to quantitate positive areas of collagen (blue in Masson's trichrome staining), elastin (black in Verhoeff's van Gieson staining), glycosaminoglycans (blue in Alcian blue staining), type I collagen and fibronectin. (A) Collagen deposition showed a reduction in GM-CSF group compared with the PBS group (B) The VFs in the GM-CSF group tended to exhibit less disorganized and tangled elastin compared with PBS group. (C) Alcian blue staining of VFs tended to reveal higher glycosaminoglycan levels in the GM-CSF group than in the PBS group. (D) Type I collagen deposition was significantly lower in the GM-CSF-treated VFs than the PBS group. (E) Quantitative analysis of fibronectin-positive areas revealed significantly less positivity in the GM-CSF-treated group than the PBS group (n = 12, *P<0.05 and **P<0.01).

Immunohistochemical analysis demonstrated that the deposition of type I collagen and fibronectin was lower in GM-CSF-treated VFs than in PBS-treated VFs (Fig. 5). These results were further confirmed by densitometric analysis using MetaMorph software (Figs. 6A∼6E).

### Effects of GM-CSF on the hVFF morphology and proliferation

**Figure 7:**
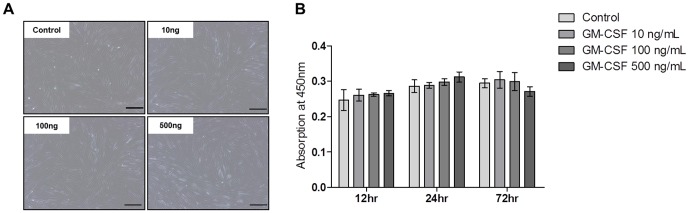
Effects of GM-CSF treatment on hVFF morphology and proliferation. (A) Phase contrast microscope images of hVFFs cultured with GM-CSF (0, 10, 100, 500 ng/mL) for 72 h. Scale bar = 200 µm. (B) Cell proliferation was measured after GM-CSF treatment at 12, 24, and 72 hrs using a CCK-8 assay. GM-CSF did not significantly alter hVFF morphology or proliferation.

hVFFs cultured in serum-free medium exhibited typical spindle-like fibroblast morphology, and showed no significant change in morphology when treated with different dosages (10–500 ng/mL) of GM-CSF over 72 hours (Fig. 7A). Proliferation of hVFFs in response to different doses of GM-CSF was assessed using the Cell Counting Kit-8 (CCK-8) and showed no significant changes over time (Fig. 7B).

### ECM production by hVFFs after GM-CSF treatment

**Figure 8:**
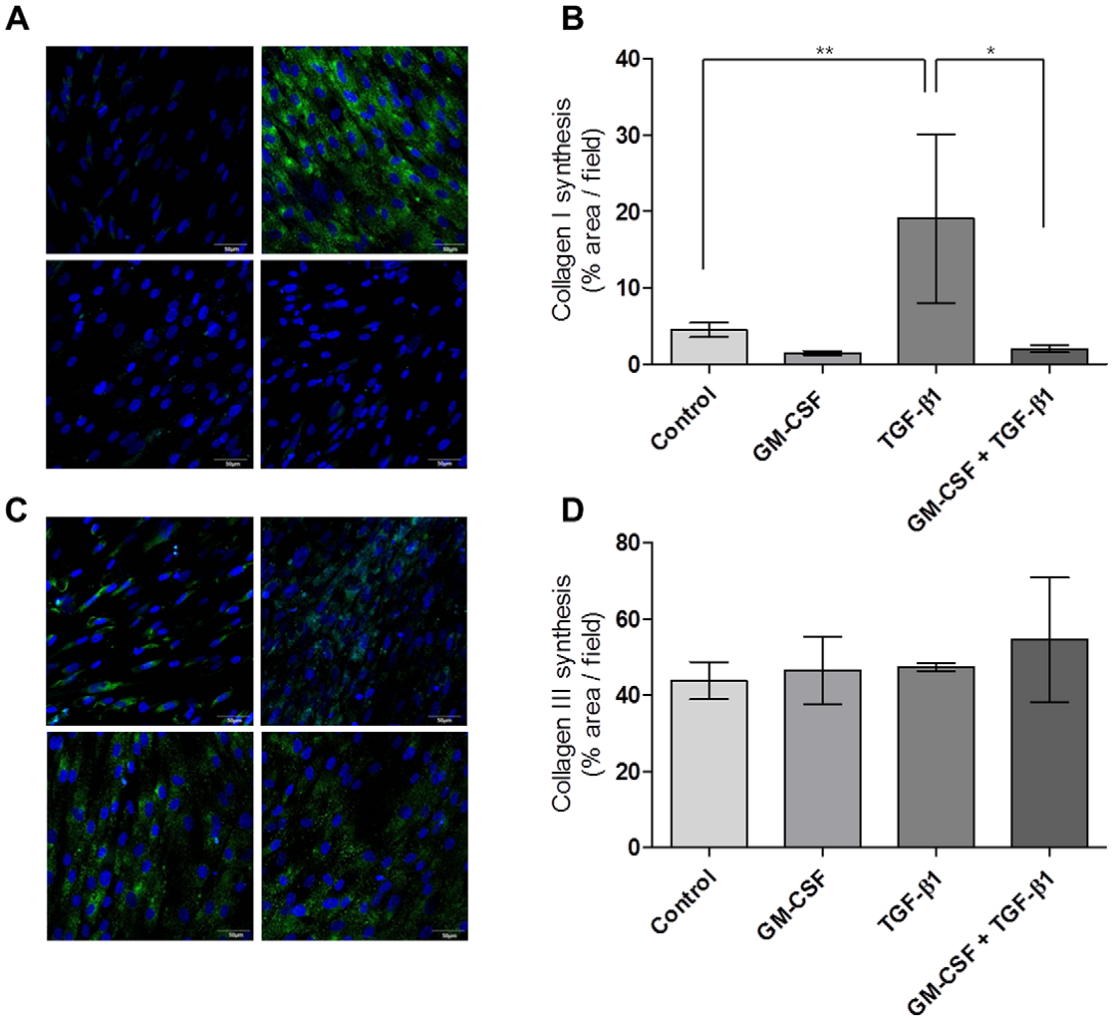
TGF-ß1-induced collagen production in hVFFs after GM-CSF treatment. (A and B) Immunocytochemistry for type I collagen revealed that GM-CSF itself did not affect the production of type I collagen in hVFFs at 72 hours after treatment. However, the TGF-ß1-induced synthesis of type I collagen significantly decreased by GM-CSF treatment (*P<0.05 and **P<0.01). (C and D) GM-CSF did not affect the production of type III collagen in hVFFs, regardless of TGF- ß1 treatment (*P*>0.05). All experiments were carried out in triplicate.

In order to assess ECM production in response to GM-CSF treatment, we first measured TGF-ß1-induced type I and III collagen accumulation in hVFFs after 72 hrs of treatment. TGF-ß1 is known to be involved during acute inflammation and neomatrix deposition following VF injury; moreover, exogenous TGF-ß1 has been shown to increase collagen secretion from hVFFs [19]. Immunocytochemistry revealed that GM-CSF did not affect type I and type III collagen (both *P*>0.05, Fig. 8) production by itself, but significantly reduced TGF-ß1-induced accumulation of type I collagen, but not type III (*P*<0.05, Figs. 8A and 8B).

**Figure 9:**
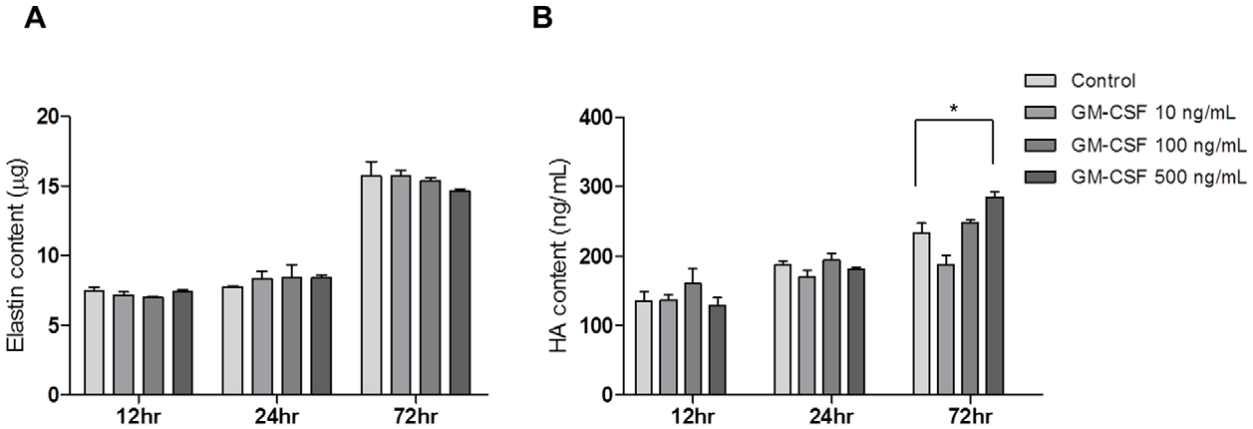
Biochemical analyses of elastin and HA synthesis in hVFFs after GM-CSF treatment. (A) The production of elastin in hVFFs was not found to be significantly dependent on GM-CSF concentration or treatment time (*P*>0.05). (B) HA levels significantly increased after 72 hours of GM-CSF treatment only at 500 ng/mL (*P<0.05). All experiments were performed in triplicate.

Subsequently, the effect of GM-CSF on the production of elastin in hVFFs and secretion of HA to the culture media was examined. The elastin content was not significantly different upon GM-CSF treatment at all concentrations and time-points tested (*P*>0.05, Fig. 9A). HA levels were significantly greater in culture media only when GM-CSF was treated at 500 ng/mL for 72 hours (*P*<0.05, Fig. 9B).

### Gene expression after GM-CSF treatment

To gain further insight into the mechanism of ECM production by GM-CSF, the expressions of genes related to ECM components and ECM production-related growth factors, such as HGF and TGF-ß1, were examined.

**Figure 10:**
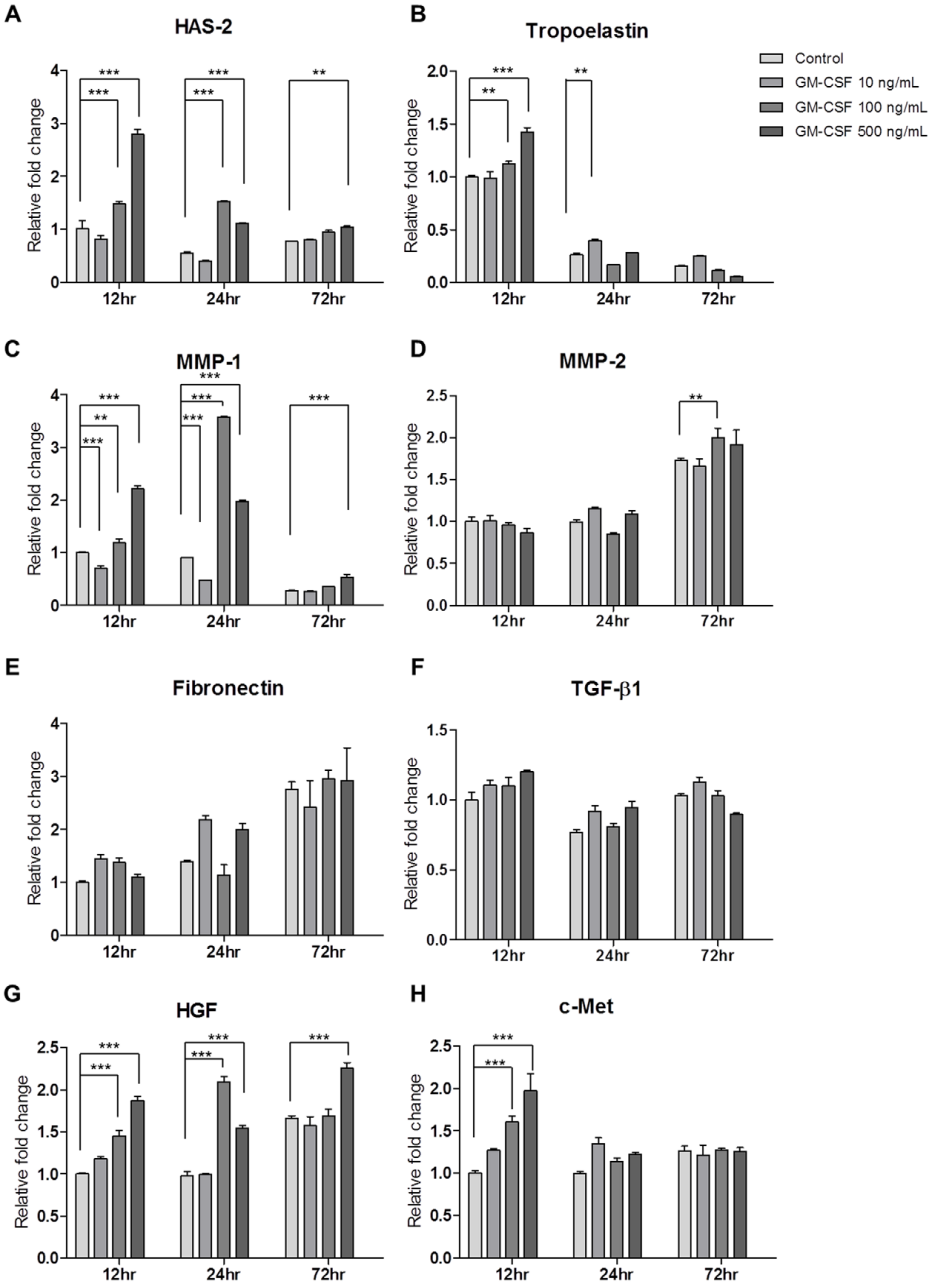
Real time PCR of gene expression in hVFFs after GM-CSF treatment. hVFFs were treated with 0, 10, 100 and 500 ng/mL of GM-CSF for 12, 24, and 72 hours. Real-time PCR showed that GM-CSF treatment increased the expression of HAS-2, tropoelastin, HGF, c-Met, and MMP-1 at different time points. All experiments were carried out in triplicate. Results were normalized versus mRNA levels of GAPDH. *P<0.05, **P<0.01, and ***P<0.001.

HAS-2 expression was significantly elevated at 12 and 24 hours upon treatment with 100 and 500 ng/mL of GM-CSF (*P*<0.001), and this increase was maintained up to 72 hours upon treatment with 500 ng/mL of GM-CSF (*P*<0.01, Fig. 10A). Tropoelastin expression was up-regulated dose-dependently by GM-CSF at 12 hours, but decreased significantly thereafter with the GM-CSF, demonstrating a negligible and varying effect (*P*<0.01, Fig. 10B). Furthermore, GM-CSF elicited the induction of MMP-1 at 12 and 24 hours (*P*<0.01), which was greatly reduced but sustained at 72 hours by treatment with 500 ng/mL of GM-CSF (*P*<0.001, Fig. 10C). The mRNA level of MMP-2 was significantly increased by treatment with 100 ng/mL of GM-CSF at 72 hours (*P*<0.01, Fig. 10D). GM-CSF also significantly up-regulated mRNA levels of HGF and c-Met at 12 hours in a dose-dependent manner, but that of HGF was only maintained high by GM-CSF at 24 and 72 hrs (*P*<0.001, Figs. 10G and 10H). In contrast, GM-CSF did not significantly affect the expression of fibronectin or TGF-ß1 at any time point (Figs. 10E and 10F). Transcriptional analyses of the expressions of type I and type III collagen mRNA were conducted after treating hVFFs with 10 ng/mL of TGF-ß1 with or without GM-CSF (10, 100 and 500 ng/mL) for 3 days. Compared with the TGF-ß1 treatment alone, co-treatment of TGF-ß1 and GM-CSF did not reduce the mRNA levels of type I or type III collagen (data not shown).

## Discussion

Wound healing is a complex process that is controlled by many growth factors and cytokines. Furthermore, multiple biological pathways are known to be activated and involved in synchronizing responses after tissue injury [20]–[22]. However, wound repair commonly leads to fibrotic healing, which results in scar formation rather than tissue regeneration, and thus, many studies have been undertaken to redirect fibrotic healing processes toward regenerative healing by modulating specific pathways.

The process of fibrotic wound healing is known to be cell- and microenvironment-specific. VF scars are common and usually even a small lesion in VF epithelium can have a significant effect on voice production. Some studies have reported that the expressions of several inflammatory cytokines and growth factors change in-line with changes in ECM production following VF injury *in vivo* [23]–[27]. Furthermore, others have suggested possible target cytokine pathways, such as interleukin-1 ß (IL-1ß), tumor necrosis factor-α (TNF-α), and prostaglandin E_2_ (PGE_2_), as candidates for the mitigation of fibrotic scar formation [28]–[30]. However, the events following VF injury have only partially been elucidated.

The present study demonstrated that GM-CSF administration can promote VF regeneration by wound remodeling following injury, and improve the functional properties of VFs, i.e. biomechanical aspects such as viscoelastic properties and mucosal waves. We speculate that these results were related to *in vivo* findings, namely reduced depositions of type I collagen and fibronectin, less disorganized elastin, and increased HA synthesis, in comparison to PBS treated controls. In our *in vitro* study, we found that GM-CSF rescued excessive collagen production and increased HA synthesis, possibly via collagen degradation due to MMP-1 activation and via HAS up-regulation.

No consensus has been reached regarding the relation between GM-CSF and fibrosis. In one study, exogenous GM-CSF administration was found to inhibit fibrosis in human *in vivo* study and to down-regulate collagen production selectively in wound fibroblasts *in vitro* [31]. However, in another study, no effect was noted following systemic injection of GM-CSF [32]. In the present study, *in vivo* collagen deposition was found to be reduced after GM-CSF treatment, but type I collagen mRNA expression was not reduced when hVFFs were in the presence of TGF-ß1 *in vitro*. Thus, fewer fibrotic changes in GM-CSF-treated VFs than in PBS-treated VFs could be attributed to collagen degradation by MMP-1 or the attenuating effect of HAS-2 on collagen synthesis in hVFFs. Accordingly, we are of the opinion that GM-CSF plays a complex role in the fibrotic process depending upon the circumstances.

HA is a non-sulfated glycosaminoglycan and exhibits extensive molecular interactions that cause it to remain entangled and hydrated in the ECM, and interestingly, HA has been reported to play an important role in determining the viscoelastic properties of VFs [33]. In this study, GM-CSF-treated hVFFs exhibited elevated HA levels, possibly due to HAS elevation. However, it remains to be determined whether treatment of hVFFs with GM-CSF alone elevates HAS-2 expression and whether this up-regulation or the secondary stimulation of HGF induces HA synthesis. The administration of GM-CSF did not appear to regulate elastin synthesis directly, although *in vivo* histologic examinations exhibited less elastin disorganization and fragmentation in scarred VFs.

GM-CSF reportedly stimulates wound repair both directly and indirectly via induction of secondary cytokines [34], [35]. In addition to the primary chemotactic effects of GM-CSF on neutrophils and macrophages that lead to inflammatory response and the promotion of wound repair, GM-CSF has been observed to cause endothelial cells and fibroblasts to secrete other growth factors in an autocrine or paracrine fashion.

In this study, GM-CSF up-regulated HGF and its membrane-spanning tyrosine kinase receptor, c-Met, but did not regulate TGF- ß1 expression. The growth factors, HGF and TGF-ß1, have been shown to play different roles in the stimulation and inhibition of ECM production by hVFFs. HGF favors HA up-regulation and collagen down-regulation, whereas TGF-ß1 increases the production of type I collagen and fibronectin, which plays a major etiologic role in chronic fibrosis [27]. Thus, alterations in the balance between HGF and TGF-ß1 are likely to cause suboptimal wound healing. HGF activity was reported to peak by day 10 following VF injury in a rabbit model [26]. In our study, HGF expression was significantly increased at 12 hours and was maintained at a high level at 24 and 72 hours by GM-CSF. Thus, we assumed that the indirect actions of HGF, induced more rapidly by exogenous GM-CSF, likely promoted the wound healing process in our model.

Our *in vitro* study has some limitations. First, hVFFs were not in their native physiologic environment and *in vitro* data cannot represent the complex interactions that exist between multiple cell types, matrices, and mediators at the sites of injury. Second, it is difficult to draw conclusions regarding optimal dosages and appropriate times of administration due to the variability of expression data obtained at particular time points following GM-CSF treatment. Although *in vivo* animal models may concur with *in vitro* data and provide biological insights of the functions of different mediators, we suggest that a transgenic and knock-out mouse model should be developed for future wound healing experiments.

### Conclusion

The results of the present study suggested that GM-CSF plays a role in wound remodeling following VF injury, and that GM-CSF either directly or indirectly exerts favorable effects such as the rescue of excessive collagen deposition and an increase in HA synthesis. Finally, our findings suggest that GM-CSF should be viewed as a potential therapeutic candidate for the prevention of VF scarring and the promotion of VF regeneration.
